# Detecting Elder Abuse in an Italian Emergency Department: A Six-Year Retrospective Study and Implications for Systematic Screening

**DOI:** 10.3390/geriatrics11040079

**Published:** 2026-07-02

**Authors:** Martina Focardi, Paola D’Onofrio, Marco Carnevali, Francesca Romana Ermini, Edoardo Orlandi, Ilenia Bianchi, Barbara Gualco, Vilma Pinchi, Beatrice Defraia

**Affiliations:** 1 Multidisciplinary Research Laboratory in Forensic Sciences (CRIME-LAB), Department of Health Sciences, University of Florence, 50134 Florence, Italy; martina.focardi@unifi.it; 2 Hospital Management Rosa Code Unit, AOUC-Careggi University Hospital, 50134 Florence, Italy; donofriop@aou-careggi.toscana.it (P.D.); carnevalim@aou-careggi.toscana.it (M.C.); 3 Santa Maria Nuova Hospital, 50122 Florence, Italy; erminifr@aou-careggi.toscana.it; 4 Department of Health Sciences, Section of Forensic Medical Sciences, University of Florence, 50134 Florence, Italy; edoardo.orlandi@unifi.it (E.O.); barbara.gualco@unifi.it (B.G.); beatrice.defraia@unifi.it (B.D.); 5 Laboratory of Personal Identification and Forensic Morphology, Department of Health Sciences, University of Florence, 50134 Florence, Italy; vilma.pinchi@unifi.it

**Keywords:** elder abuse, elder mistreatment, physical abuse, domestic violence, elder neglect, emergency department screening, passive detection

## Abstract

**Background/Objectives:** Elder abuse remains a significantly underreported public health issue. The study examines how elder abuse is detected through a passive, suspicion-based case-finding pathway in an Italian university hospital emergency department (ED) and what the findings imply for improving systematic screening. **Methods:** This retrospective study analyzed elder abuse cases accessed at Careggi University Hospital ED (Florence, Italy) from 2017 to 2022. Eligible patients were aged ≥65 years and had suspected or confirmed elder abuse identified through Rosa Code protocol activation, abuse-related ICD-10 codes, and forensic consultation records. Two investigators independently reviewed eligible charts using predefined inclusion criteria and a standardized data-extraction form. Missing or unclear documentation was quantified descriptively, and no imputation was performed. **Results:** Sixty-seven elder abuse cases were identified during the six-year period, corresponding to a reported detection rate of 0.8% among the records screened for this study (mean: 11.2 cases/year). All eligible cases were captured through Rosa Code activation; ICD-10 and forensic-record searches did not identify additional cases. The majority of victims were women (76.1%), with a mean age of 75.5 years, and 76.1% had documented comorbidities. Physical abuse was the most common form (61.2%), predominantly perpetrated by family members (93.8%) within the victim’s home (64.2%). Head and neck injuries were most frequent (43.3%). A notable 50% decline in reported cases occurred during the COVID-19 pandemic. Despite law enforcement notification in 78% of cases, 65.7% of patients were discharged home. **Conclusions:** The study’s detection rate (<1%) falls critically short of international benchmarks (3–5%), underscoring urgent need for systematic screening using validated tools and staff training and multidisciplinary safeguarding pathways in Italian emergency departments.

## 1. Introduction

Over the last two decades, elder abuse—also referred to as elder mistreatment or elder maltreatment—has increasingly captured the attention of both the scientific and judicial/legal communities. This growing focus stems primarily from the rapidly ageing global population. Current demographic data from the World Health Organization (WHO) and the United Nations indicate a significant increase in life expectancy and a consequent rise in the number of people aged 60 and over. Specifically, by 2030, one in six people worldwide will be aged 60 or older, representing approximately 16% of the global population [1,2].

Based on these demographic trends, the burden of elder abuse is expected to rise dramatically. Community-based estimates vary by setting, abuse definition, and assessment method. Yon et al. reported a pooled past-year prevalence of 15.7%, approximately one in six community-dwelling older adults, whereas more recent meta-analytic evidence reveals that the global prevalence of elder abuse is substantially higher than previously estimated, with a pooled prevalence of 27.6% (95% CI: 23.5–31.6%) across all types of abuse [3,4]. This translates to approximately one in four older adults experiencing some form of abuse annually in community settings [5,6]. Breaking down by abuse type, the prevalence rates are: 20.9% for emotional/psychological abuse, 19.3% for neglect, 11.7% for financial abuse, 11.0% for verbal abuse, 7.9% for physical abuse, 1.5% for sexual abuse [3]. These figures suggest that by 2050, hundreds of millions of elderly individuals worldwide may be at risk of experiencing abuse.

Beyond demographic concerns, elder abuse has garnered scientific attention due to its severe psychological, physical and social consequences. Elder abuse is associated with increased morbidity and mortality, with victims experiencing higher rates of depression, anxiety, post-traumatic stress disorder, and premature death [7,8,9,10]. Multiple systematic reviews document associations between elder abuse and serious physical harms and long-term psychological consequences, though consistent global estimates for hospitalization and mortality rates attributable specifically to elder abuse remain limited [5,7,11].

From a public health perspective, understanding the risk factors associated with elder abuse is crucial for prevention. A comprehensive understanding of this phenomenon can facilitate the development of effective interventions to prevent the occurrence and proliferation of abuse. Recent systematic reviews have identified a recurrent set of risk factors across settings, including individual vulnerabilities (age, dependency, cognitive impairment), caregiver and relational factors (caregiver stress, poor mental health, substance misuse), and structural drivers (social isolation, institutional residence) [3,5,12,13].

Emergency departments (EDs) represent critical frontline settings for identifying and intervening in cases of elder mistreatment [14,15], as acute presentations may represent the only opportunity for isolated older adults to access healthcare outside the home environment [14].

However, ED-based detection remains challenging due to multiple factors, including time constraints, lack of standardized screening protocols, insufficient training of healthcare professionals, and the complex presentation of abuse in older adults [16,17,18,19]. Furthermore, elder abuse often co-occurs with other geriatric syndromes such as cognitive impairment, functional decline, and multiple comorbidities, making recognition more difficult [20,21,22,23,24].

Several ED studies have examined elder-abuse screening and detection. Reported detection rates vary widely, from less than 1% with passive case-finding or administrative coding to approximately 3–7% when systematic screening tools, structured assessment, or direct questioning are used. The most relevant findings suggest that structured ED workflows, staff training, direct questioning when clinically appropriate, and multidisciplinary follow-up increase identification compared with reliance on clinical suspicion alone [25,26,27,28,29]. However, evidence from European and Southern European EDs remains limited, and Italian data remain scarce. Furthermore, Italy’s universal healthcare system and mandatory reporting laws create a distinct legal and institutional context compared to other countries [30].

The COVID-19 pandemic has further complicated the landscape of elder abuse detection and intervention. Lockdown measures, social isolation, increased caregiver stress, and reduced access to healthcare and social services have been associated with increased risk of elder mistreatment [27,31,32]. Simultaneously, pandemic-related disruptions to healthcare systems may have reduced opportunities for detection and reporting [33,34]. Understanding pandemic impacts on elder abuse detection is essential for strengthening resilience of future screening programmes.

In Italy, the Rosa Code (Codice Rosa) protocol was established in Tuscany in 2010 as a standardized multidisciplinary response pathway for victims of violence, including elder abuse [35]. The protocol mandates coordinated involvement of emergency physicians, forensic consultants, social workers, and law enforcement when abuse is suspected. However, Rosa Code activation relies on clinical suspicion rather than systematic screening, and its effectiveness for elder abuse detection has not been rigorously evaluated [36].

In the Italian context, suspected violence against an older person may trigger both clinical safeguarding actions and legal reporting duties. Health professionals are required to report cases that may involve offences prosecutable ex officio when they become aware of them while providing care; in public hospital settings, reporting is generally directed to the Judicial Authority or judicial police/law enforcement, while social services are involved for protection, placement, and community follow-up rather than criminal investigation. This legal framework differs from adult protective services models used in some countries and is relevant when interpreting ED referral pathways and police notification rates [37].

This retrospective study aimed to examine how suspected or confirmed elder abuse was detected in an Italian university hospital ED under a passive, suspicion-based case-finding pathway. Specifically, we sought to: (1) describe case-identification routes through Rosa Code activation, abuse-related ICD-10 codes, and forensic consultation records; (2) characterize the clinical, forensic, and safeguarding features of detected cases; and (3) identify practical implications for systematic elder-abuse screening in Italian EDs. Hospital admission was analyzed only as an exploratory secondary outcome.

## 2. Materials and Methods

### 2.1. Study Design and Setting

This retrospective observational study was conducted at the Emergency Department of Careggi University Hospital, a tertiary care academic medical centre in Florence, Italy. Careggi University Hospital serves as the primary referral centre for the Tuscany region, with an ED that manages approximately 100,000 visits annually. Administrative ED data indicated that older adults (≥65 years) accounted for approximately 27,000 annual visits before the pandemic and approximately 20,000 annual visits during 2020–2021. This study was conducted in accordance with the Declaration of Helsinki and was approved by the local ethics committee (Protocol Code: Approval No. 20160118), and informed consent was waived due to the retrospective nature of the study and use of de-identified data. All data were stored securely in password-protected databases accessible only to authorized research personnel. Patient identifiers were removed and replaced with unique study codes. The research team followed strict protocols to maintain confidentiality throughout data collection, analysis, and reporting.

### 2.2. Study Population and Eligibility Criteria

We included all patients aged ≥65 years presenting to the ED between 1 January 2017 and 31 December 2022 with suspected or confirmed elder abuse. Cases were identified through three sequential sources: (1) Rosa Code protocol activation documented in ED records, (2) ICD-10 diagnostic codes related to abuse, assault, or maltreatment (T74.0–T74.9, T76.0–T76.9, Y07), and (3) manual review of forensic consultation records. Records identified by any of these routes were de-duplicated, and two investigators (MF, PD) independently reviewed the charts to confirm whether they met the study definition of elder abuse [38]. Discrepancies were resolved through consensus discussion with a third investigator (BD).

Sample was selected based on the following inclusion and exclusion criteria:

*Inclusion Criteria*

- Age ≥ 65 years at the time of ED presentation.
- Documented or suspected domestic violence, abuse, or mistreatment by a caregiver, family member, cohabitant, or trusted person.
- Complete medical record available for review.
- Any sex or gender.

*Exclusion Criteria*

- Age < 65 years.
- No documentation supporting suspected elder abuse, domestic violence, or mistreatment.
- Other forms of violence not classified as domestic violence (e.g., hate crimes, stranger assault, sexual assault by non-intimate partners, accidental injuries).
- Cases involving only self-neglect without evidence of mistreatment by others.
- Incomplete or unavailable medical records.

### 2.3. Case Identification and Chart Review

In the final review, all included cases had been captured through Rosa Code protocol activation; the ICD-10 and forensic-record searches did not identify additional eligible cases beyond those already captured by Rosa Code.

Case-identification flow: Older-adult ED records during 2017–2022 → records flagged by Rosa Code activation, abuse-related ICD-10 codes, or forensic consultation review → de-duplicated records reviewed independently by two investigators → final analytic sample of 67 confirmed/suspected elder abuse cases. This flow clarifies that the criteria listed below were applied to records identified through the three screening sources, not to every ED record manually.

### 2.4. Definition and Classification of Elder Abuse

Elder abuse was defined as intentional or negligent acts by a caregiver, family member, or trusted person that caused harm or created serious risk of harm to an older adult [6]. In this study, the term domestic violence was used operationally to indicate violence or mistreatment occurring within domestic, family, cohabiting, caregiving, or other trusted relationships. Accordingly, physical, psychological/emotional, financial, and neglectful acts could fall within the study definition when perpetrated by a family member, caregiver, or other trusted person. Assaults by strangers, hate crimes, and accidental injuries were excluded.

Cases were included if documentation supported reasonable suspicion of abuse based on

- Patient or witness disclosure;
- Injury patterns inconsistent with reported mechanism;
- Unexplained injuries or delays in seeking care;
- Evidence of neglect or self-neglect;
- Forensic consultant assessment indicating probable abuse.

### 2.5. Rosa Code Protocol and Multidisciplinary Pathway

The Rosa Code protocol, implemented at Careggi Hospital in 2010, provides a standardized multidisciplinary response pathway for suspected violence victims [35]. When elder abuse is suspected, ED staff activate the Rosa Code protocol, triggering immediate forensic medicine consultation, comprehensive injury documentation including photographs and body diagrams, biological sample collection when indicated, social work assessment, risk assessment using standardized tools, and coordination with law enforcement and adult protective services. The protocol emphasizes patient safety, evidence preservation, and coordinated intervention.

### 2.6. Data Collection, Study Variables and Outcome

Data were extracted using a standardized data collection form. The variables collected included demographic characteristics, such as age, sex, nationality, living situation, marital status, and presence of caregivers; medical history, including comorbidities, medication use, cognitive status when documented, functional status, and previous episodes of abuse; and abuse-related characteristics, including type of abuse, perpetrator relationship, location of the event, mechanism of injury, duration of abuse, and prior abuse history. Clinical variables included chief complaint, triage acuity level, time from injury to ED presentation, injury type, anatomical distribution, injury severity, diagnostic tests, imaging studies, and specialist consultations. Information on DA5 risk assessment scores, when available, was also collected. Outcomes included ED disposition, hospital admission, discharge destination, law enforcement notification, social work consultation, referral to social or protective services, and follow-up arrangements.

Abuse types were classified according to the information documented in the ED and forensic records and according to the literature [3,23,38,39]. The intentional use of physical force that may result in injury, physical pain, bodily impairment, or death was considered. Psychological or emotional abuse included verbal aggression, threats, humiliation, intimidation, coercive behaviour, or isolation causing emotional distress. Neglect was defined as failure by a caregiver or trusted person to provide necessary care, assistance, supervision, or protection. Financial exploitation included unauthorized or improper use of the older person’s money, property, or resources. Sexual abuse was defined as any non-consensual sexual contact. When more than one type of abuse was documented, multiple abuse categories were recorded for the same case.

Injury severity was classified as minor, moderate, or severe on the basis of clinical documentation. Minor injuries included superficial lesions that did not require specific medical intervention. Moderate injuries were those requiring medical evaluation or treatment but not hospital admission. Severe injuries were defined as injuries requiring hospital admission or associated with significant functional impairment.

Pandemic periods were defined as pre-pandemic (January 2017–December 2019), pandemic (January 2020–December 2021), post-acute pandemic (January 2022–December 2022).

Cognitive impairment was documented based on clinical assessment, prior diagnoses, or family report, recognizing that systematic cognitive screening was not routinely performed.

Because this was a retrospective chart-review study, documentation completeness varied across variables. Variables not documented in the medical record were coded as missing or as ‘not documented/unclear/not specified’ when clinically meaningful; absence of documentation was not interpreted as absence of the finding. For variables with incomplete documentation, the available denominator and the number of missing/unclear records are reported in the Supplementary Material—Table S1. No imputation was performed.

### 2.7. Statistical Analysis

Descriptive statistics summarized patient characteristics, abuse patterns, and outcomes. Continuous variables were reported as means with standard deviations (SDs) or medians with interquartile ranges (IQRs) depending on distribution. Categorical variables were reported as frequencies and percentages.

Temporal trends were analyzed using chi-square tests comparing case frequencies across pre-pandemic, pandemic, and post-acute pandemic periods. Fisher’s exact test was used when expected cell counts were <5.

Hospital admission was examined only as an exploratory secondary outcome because admissions were infrequent. To avoid over-interpretation of unstable estimates, the detailed multivariable admission model is available in Supplementary Material—Table S2).

All statistical tests were two-tailed with significance set at *p* < 0.05. Analyses were performed using STATA version 17.0 (StataCorp., College Station, TX, USA).

## 3. Results

### 3.1. Case Identification and Temporal Trends

**Figure 1:**
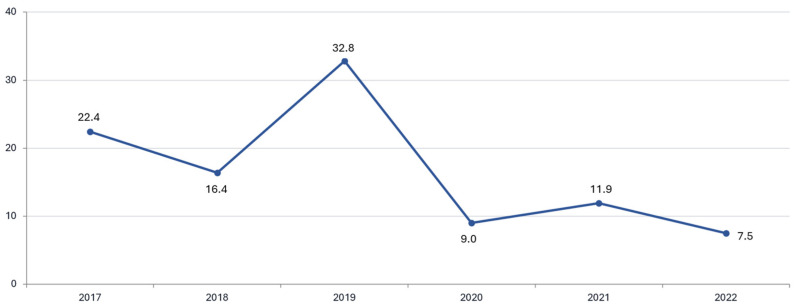
Annual distribution of elder abuse cases, 2017–2022. Note: The reduction in cases during 2020–2021 corresponds to the COVID-19 pandemic period.

A total of 67 cases of elder abuse were identified during the six-year study period, representing a mean of 11.2 cases per year (range: 5–22 cases/year). Figure 1 illustrates the temporal distribution of cases. A notable decline in detected cases was observed during the COVID-19 pandemic years (2020–2021), with only 6 and 8 cases identified in 2020 and 2021, respectively, compared to a pre-pandemic average of 13 cases per year (2017–2019).

All 67 cases (100%) were identified through Rosa Code protocol activation, indicating that clinical suspicion-based detection was the sole identification pathway. No additional cases were identified through ICD-10 coding or chart review beyond those already captured by Rosa Code activation. This finding indicates that the observed cohort reflects a passive, suspicion-based pathway rather than systematic ED screening.

### 3.2. Demographic and Clinical Characteristics of Victims

The mean age of victims was 75.5 years (SD = 7.8 years; range: 65–94 years), with the majority (58.2%) in the 65–74 age group. Female victims predominated, accounting for 76.1% (*n* = 51) of cases. The vast majority of victims were Italian nationals (91.0%, *n* = 61), with only 9.0% (*n* = 6) being foreign nationals. Most victims lived in private homes (89.6%), either alone (31.3%) or with family members (58.3%). Only 10.4% resided in assisted living facilities at the time of the abuse.

Medical comorbidities were present in 76.1% (*n* = 51) of victims while 23.9% (*n* = 16) had either negative pathological history or unspecified medical background. The most common comorbidities were hypertension (20.9%, *n* = 14), diabetes mellitus (13.4%, *n* = 9), cognitive impairment or dementia (9.0%, *n* = 6), and cardiovascular disease (7.5%, *n* = 5).

A heterogeneous group of other conditions—including chronic obstructive pulmonary disease, atrial fibrillation, visual impairment, hearing loss, and osteoporosis—collectively represented 34.3% of cases (*n* = 23).

### 3.3. Abuse Characteristics: Types of Abuse

Among the 67 cases, the specific type of abuse was documented in 52 cases (77.6%), whereas in 15 cases (22.4%) the available records did not permit classification into a specific abuse category. Because more than one type of abuse could be recorded for the same patient, the categories were not mutually exclusive. Physical abuse was the most frequently documented form, occurring in 41 cases (61.2%), followed by psychological or emotional abuse in 16 cases (23.9%), financial abuse or exploitation in 6 cases (9.0%), and neglect in 4 cases (6.0%). No cases of sexual abuse were documented. Multiple forms of abuse were recorded in 10 cases (14.9%) (Table 1).

Among the 41 cases of documented physical abuse, natural blunt force (kicks, punches, slaps) was the most common mechanism of injury, accounting for 68.3% of cases (*n* = 28). Blunt objects were used in 17.1% of cases (*n* = 7), while combined use of natural blunt force and blunt objects occurred in 12.2% (*n* = 5). In one case (2.4%), natural blunt force was combined with an asphyxiating method.

Contusions and hematomas were the most common injuries documented, affecting 35.8% of all cases (*n* = 24). Abrasions were documented in 13.4% of cases (*n* = 9), while fractures occurred in 6.0% (*n* = 4). Notably, 13.4% of victims (*n* = 9) presented without visible external injuries despite documented abuse. Other injury types not otherwise specified were documented in 31.3% of cases (*n* = 21).

Among the 24 victims who presented with bruising or hematomas, 4 cases (16.7%)—2 males and 2 females—were documented as taking anticoagulant and/or antiplatelet medications, which may have contributed to the severity or visibility of the contusions.

The head and neck region was the most frequently injured anatomical area, accounting for 43.3% of documented injuries (*n* = 29). Upper extremities were affected in 31.3% of cases (*n* = 21), lower extremities in 16.4% (*n* = 11), thorax/chest in 6.0% (*n* = 4), and other regions (abdomen, pelvis, back) in 9.0% (*n* = 6) (Table 2).

### 3.4. Perpetrator Relationship and Abuse Setting

Perpetrator relationship was documented in 64 of 67 cases (95.5%), whereas it was not documented in 3 cases (4.5%). Among cases with documented perpetrator information, abuse was predominantly intra-familial. Family members accounted for 60 of 64 documented cases (93.8%). Adult children were the most frequently identified perpetrators (*n* = 29, 45.3% of documented cases), followed by spouses or partners (*n* = 23, 35.9%) and other family members (*n* = 8, 12.5%). The remaining documented cases involved non-family or otherwise trusted persons (*n* = 4, 6.3%). These findings indicate that, in this cohort, elder abuse detected in the ED occurred mainly within domestic and caregiving relationships rather than in encounters with strangers.

The abuse setting was documented in 51 of 67 cases (76.1%) and was not specified in 16 cases (23.9%). Considering the whole cohort, the victim’s home was the most frequently documented setting, accounting for 43 cases (64.2% of all cases; 84.3% of cases with documented setting). Other settings were less frequent and included the home of a family member in four cases (6.0% of all cases), public spaces in three cases (4.5%), and the hospital or Emergency Department in one case (1.5%). Abuse was documented as chronic or ongoing in 52.2% of cases and as acute or isolated in 47.8%. Precipitating or contextual factors recorded in the medical records included caregiver stress, financial disputes, substance misuse by the perpetrator, and mental health issues of the perpetrator.

### 3.5. Clinical Presentation and ED Assessment

Emergency medical services (ambulance) represented the most common mode of arrival across all age cohorts (46.3%, *n* = 31), followed by self-presentation (26.9%, *n* = 18). Police escort accounted for 14.9% (*n* = 10), while other modes comprised 11.9% (*n* = 8). Mode of arrival should not, however, be interpreted as equivalent to the source of referral or to the individual who first suspected abuse.

None of the included cases entered the study cohort through a documented referral for suspected elder abuse from a primary care physician, continuity-of-care physician, or another healthcare institution. Thus, no case was identified through a documented physician-initiated referral before arrival at the ED. This finding should be interpreted cautiously because referral information was obtained retrospectively from clinical records and may have been incompletely documented. Nevertheless, it indicates that the cases captured in this study were not primarily identified through formal referrals from community or hospital-based physicians, further supporting the predominantly passive and suspicion-based nature of the detection pathway.

The most frequent presenting complaint was trauma or injury (*n* = 35, 52.2%), followed by pain (*n* = 14, 20.9%), general malaise or non-specific symptoms (*n* = 10, 14.9%), and psychological distress (*n* = 8, 11.9%). These findings indicate that most detected cases presented with an overt physical or symptomatic complaint rather than specifically requesting assistance for elder abuse.

Information regarding previous episodes of abuse was available in 50 of the 67 cases (74.6%) and was missing or unclear in 17 cases (25.4%). Among the 50 cases with available information, previous abuse was documented in 19 patients (38.0%), whereas 31 patients (62.0%) had no previous episodes documented. Of the 19 patients with a positive history, 14 had experienced multiple previous episodes, 3 reported a single previous episode, and 2 had a documented history of previous abuse without specification of the number of episodes). The substantial proportion of records with missing or unclear information should be considered when interpreting the frequency of recurrent abuse.

Imaging examinations were performed in 41 cases (61.2%). These included plain radiography in 29 cases (43.3%), computed tomography in 17 cases (25.4%), and ultrasonography in 5 cases (7.5%); more than one imaging modality could be performed in the same patient. Laboratory investigations were obtained in 37 cases (55.2%). Social work consultation was documented in 48 cases (71.6%), psychiatric consultation in 10 cases (14.9%), and forensic medicine consultation in 7 cases (10.4%).

The DA5 risk-assessment tool was introduced into the Rosa Code pathway in April 2018 and was therefore potentially available for only 28 of the 67 patients included in the study (41.8%). Among these 28 cases, a score of 2 was the most frequent single category (*n* = 11, 39.3%), but it did not represent the majority of assessments. Six patients scored 3 (21.4%) and one scored 4 (3.6%). In the remaining 10 cases (35.7%), the score was recorded as 0–1. (Table 3).

### 3.6. External Agency Notification and Referral Pathways

Law enforcement (police) was notified in the majority of cases (78.0%, *n* = 52) and a formal report was submitted to the Judicial Authority in the same cases. Referrals to social services were less frequent (15.0%, *n* = 10), and anti-violence centres were contacted in 7.0% of cases (*n* = 5). Adult protective services were contacted in 4.5% of cases (*n* = 3).

### 3.7. ED Disposition and Clinical Outcomes

The majority of victims were discharged home following evaluation and treatment (*n* = 44, 65.7%) and were referred to social services, anti-violence centres and adult protective services. Hospital admission was necessary in six cases (9.0%). Transfer to observation unit occurred in eight cases (11.9%), while other dispositions (including transfer to other facilities) accounted for 13.4% (*n* = 9). No cases of patients leaving against medical advice (AMA) or death in the ED were documented during the study period.

### 3.8. Impact of COVID-19 Pandemic

Comparing the pre-pandemic period (2017–2019) with the pandemic period (2020–2022), we observed a 39.6% reduction in detected cases during the pandemic years (19 cases vs. 48 cases).

To contextualize this reduction, we obtained overall ED visit data for older adults (≥65 years) during the study period. ED visits by older adults decreased by approximately 25% during 2020–2021 compared to 2017–2019 (from ~27,000 to ~20,000 annual visits), indicating that the 50% reduction in abuse case detection exceeded the overall decrease in ED utilization. This suggests pandemic-specific disruption of abuse detection mechanisms beyond general ED volume changes.

Comparison of victim characteristics across pandemic periods revealed no significant differences in age, sex, abuse types, injury severity, or outcomes (all *p* > 0.05), suggesting that detected cases represented similar clinical presentations throughout the study period, but detection sensitivity decreased during the pandemic. No significant differences were observed in victim demographics, abuse types, or perpetrator characteristics between the two periods.

As an exploratory analysis, hospital admission occurred in six cases (9.0%). Table S2 in Supplementary Material shows that severe injury could be identified as the factor most strongly associated with hospital admission. Because of the very small number of admission events, no definitive conclusions regarding independent predictors could be drawn, and the detailed analysis is reported in Supplementary Table S2.

### 3.9. Comparison with International Studies

Demographic patterns showed remarkable consistency across countries. Female predominance ranged from 55.7 to 76.1%, with most studies reporting approximately 70–75% female victims [15,27,40,41,42,43,44]. Mean ages were similar (75–78 years) across studies. Physical abuse was the most commonly detected type in most studies, though the Swiss study reported higher rates of neglect detection [41].

Table 4 compares findings from this study with recent international ED-based elder abuse studies. Detection rates varied substantially across studies, ranging from 0.013% (passive case-finding in U.S. administrative data) [45] to 7.0% (systematic screening in U.S. academic ED) [15]. Studies employing validated screening tools consistently identified higher rates (2.9–7.0%) [15,27,41,42,43,44] compared to passive case-finding approaches (<1%) [45].

Family perpetrators predominated across all studies (>90%), with adult children and spouses as the most common perpetrators. This cross-cultural consistency supports generalizability of abuse dynamics despite varying healthcare systems and cultural contexts.

## 4. Discussion

Elder abuse remains a widespread yet largely unacknowledged problem across healthcare systems worldwide. Recent international evidence confirms that at least one in six older adults experiences some form of abuse, with substantial geographic and methodological variation in reported prevalence rates [4,6]. The most current European synthesis reported that 15.4% of community-dwelling older adults experienced abuse in the past year, with institutional rates reaching up to 33% in some settings [48]. These figures represent a critical public health burden that demands enhanced detection and intervention strategies, especially in acute care settings such as emergency departments (EDs).

This six-year retrospective study from an Italian university hospital emergency department identified 67 cases of elder abuse among patients aged ≥65 years, representing a mean detection rate of 11.2 cases per year. Our findings are consistent with international evidence suggesting that elder abuse remains substantially under-detected in healthcare settings [1].

Three principal findings emerge. First, all cases were identified through Rosa Code activation, while ICD-10 and forensic-record searches did not identify additional cases, showing that detection depended entirely on clinical suspicion and protocol activation rather than systematic screening. Second, the detection rate was substantially lower than rates reported in ED studies using structured screening or direct questioning, supporting the view that passive case-finding misses many older victims. Third, the demographic pattern, abuse types, perpetrator relationships, and injury distribution were broadly consistent with international findings, supporting the relevance of international screening evidence while underscoring the need for Italian contextual adaptation [4,15,40,41,44,49].

In addition, the decline in detected cases during the COVID-19 pandemic exceeded the reduction in overall ED use by older adults, suggesting that passive detection pathways may be especially vulnerable to disruption during public health emergencies [50,51]. The exploratory hospital-admission analysis has been de-emphasized because it is not central to the screening-focused aim of this paper.

A critical finding from the recent literature is that elder abuse prevalence detected in emergency department settings is substantially lower than community estimates, likely reflecting significant under-detection rather than true lower prevalence [52]. A recent prospective Swiss ED screening study of 1010 consecutive patients aged ≥65 years reported a positive screen rate of only 2.9% using the ED Senior AID tool [41], markedly lower than the 15.4% community prevalence documented in other studies [4].

This discrepancy underscores the challenges inherent in ED-based detection and suggests that our observed case series of 67 cases over six years (averaging 11.2 cases per year) likely represents only a fraction of elder abuse victims presenting to our facility. Studies highlight multiple factors that contribute to this under-detection in emergency settings, including limited time pressures in busy EDs, poor linkage to adult protective services, inconsistent prehospital guidance [52,53]. Additionally, due to the type of abuse—often physical—that leads to presentation in the ED, other forms of abuse, which are more prevalent among older adults, tend to escape detection within this clinical setting.

In our cohort, 13.4% of victims presented without visible external injuries despite suspected or confirmed abuse. This finding should be interpreted alongside Yonashiro-Cho et al., who reported injuries at assessment in 79.0% of physically abused older adults, meaning that approximately 21.0% had no injuries detected at the time of evaluation. Their study also found that abused older adults more often had upper-extremity ecchymoses, abrasions, or tenderness and head/neck/maxillofacial ecchymoses or tenderness, which is consistent with the frequent head/neck and upper-extremity involvement observed in our cohort [54].

Healthcare professionals consistently report feeling poorly trained in elder abuse recognition and cite knowledge deficits and uncertainty about reporting processes as major obstacles [55].

International evidence demonstrates that systematic screening dramatically increases detection rates. The Canadian multicenter study detected abuse in 5.1% of older ED patients using direct questioning [39]. The Swiss study identified 2.9% using the ED Senior AID tool [41]. Even in U.S. Veterans Affairs settings with high-risk populations, systematic screening detected 2.0–3.6% positive cases [48]. In contrast, passive approaches relying on clinical suspicion or ICD coding identify <1% of cases [45], consistent with our findings. Returning to the international literature introduced in the Introduction, our <1% detection rate contrasts sharply with rates of 2.9–5.1% achieved with validated screening tools, confirming that passive case-finding systematically misses less visible or less stereotypical cases.

The Canadian direct-questioning approach may be useful in cognitively intact and communicative patients; however, its routine application in older ED populations may be challenging because cognitive impairment, delirium, dementia, sensory deficits, acute illness, fear of retaliation, and caregiver presence are common. Direct questioning should therefore be embedded within a broader triangulated ED detection strategy. Such triangulation should integrate patient disclosure, witness or caregiver accounts, inconsistencies between the reported mechanism and clinical findings, injury pattern and anatomical distribution, prior ED visits, forensic documentation, social-work assessment, law-enforcement information, and available community or social-service records.

This detection gap has profound implications. Elder abuse is associated with increased mortality (3-fold higher risk), accelerated functional decline, depression, and healthcare costs exceeding $5 billion annually in the United States alone [8]. Each missed case represents a lost opportunity for intervention, protection, and prevention of escalating harm. Our findings provide compelling evidence for the urgent need to implement systematic screening in Italian EDs using validated tools adapted to the Italian healthcare context.

Despite the low detection rate, the characteristics of detected cases closely mirror international patterns, supporting cross-cultural generalizability of abuse dynamics. The predominance of female victims (76.1%), family perpetrators (93.8%), and home-based abuse (64.2%) observed in our cohort mirrors patterns documented in systematic reviews and multi-national studies [4,56]. This pattern likely reflects multiple factors including women’s longer life expectancy, potentially greater vulnerability due to physical frailty, possible gender differences in willingness to report abuse, and historical patterns of domestic violence that may continue into older age. However, this finding must be interpreted cautiously, as male victims may face particular barriers to disclosure due to stigma and traditional gender role expectations [57].

The mean age of 75.5 years and high prevalence of comorbidities (76.1%), particularly hypertension, diabetes, and cognitive impairment, are consistent with established risk profiles identifying frailty, functional dependence, and cognitive decline as key vulnerability factors [21]. Our finding that 9.0% of victims had documented cognitive impairment likely represents substantial under ascertainment, as cognitive screening is not routinely performed in ED settings and mild-to-moderate impairment may not be documented in acute care records. Recent evidence confirms that people living with dementia (PLWD) face significantly elevated risk of all forms of abuse [58]. The Swiss ED screening study found that patients who screened positive for elder abuse were more severely cognitively impaired than those screening negative [41], corroborating this association in an acute care setting.

Physical abuse predominated in our sample (61.2%), with head and neck injuries being most common (43.0%), findings that correspond with the forensic literature documenting these anatomical regions as frequent targets in intimate partner and elder abuse contexts [59].

Our data documenting that 93.8% of identified perpetrators were family members—with adult children (45.3%) and spouses (35.9%) being most common—is consistent with recent evidence reporting that approximately 60% of elder abuse cases involve family perpetrators, with two-thirds being adult children or spouses. The finding that 64.2% of abuse incidents occurred in the victim’s home aligns with extensive literature documenting the domestic setting as the predominant location for elder abuse [10,60].

Female predominance, family perpetrators and physical abuse as the most common type show remarkable consistency across diverse healthcare systems and cultural contexts.

This consistency is reassuring for several reasons. First, it suggests that validated screening tools developed in North American settings may be applicable to Italian populations with appropriate cultural adaptation and translation. Second, it indicates that training materials and clinical guidelines can draw on international evidence while tailoring implementation strategies to local contexts. Third, it supports the hypothesis that abuse dynamics reflect universal patterns of power, dependency, and vulnerability rather than culture-specific phenomena, though cultural factors certainly influence disclosure, help-seeking, and intervention responses [61].

Several features of the Italian healthcare and legal context merit discussion. Italy’s universal healthcare system provides comprehensive coverage for older adults, potentially facilitating access to ED services compared to countries with fragmented insurance systems. However, traditional Italian family structures emphasizing intergenerational caregiving and filial responsibility may create barriers to disclosure, as victims may fear family disruption, loss of caregiver support, or bringing shame to the family [62].

Italy’s mandatory reporting laws require healthcare professionals to report suspected elder abuse to law enforcement and Judicial Authority [30]. Our high police notification rate (77.6%) reflects compliance with this legal obligation. However, mandatory reporting remains controversial, with concerns that it may deter victims from seeking care or disclosing abuse if they fear legal consequences for family members [63]. Future research should examine whether mandatory reporting affects disclosure rates and patient outcomes in the Italian context.

The Rosa Code protocol represents a strength of the Tuscany system, providing standardized multidisciplinary response pathways for violence victims [35]. However, our findings highlight a critical limitation: Rosa Code activation depends on clinical suspicion, which captures only the most obvious cases. Integration of systematic screening tools into the Rosa Code pathway could substantially enhance detection while preserving the protocol’s strengths in coordinated response and evidence preservation. Critically, no referrals in our cohort originated from physicians; all cases were flagged by nursing staff, social workers, or arrived via law enforcement or self-referral. This represents a significant gap: physicians are the primary clinical contact in the ED, and their failure to initiate safeguarding referrals underscores the need for targeted physician training in recognizing elder abuse indicators.

The marked reduction in elder abuse case detection during the COVID-19 pandemic (2020–2021) exceeded the 25% decrease in overall ED utilization by older adults, indicating pandemic-specific disruption of detection mechanisms beyond general volume changes. This finding aligns with international reports of reduced domestic violence reporting during pandemic lockdowns despite concerns about increased abuse risk [64,65,66].

The COVID-19 pandemic created a “perfect storm” for elder abuse, with multiple converging risk factors including prolonged social isolation, economic instability, heightened caregiver stress, and disrupted access to protective services [25].

Contemporary reports documented substantial increases in elder abuse risk during 2020–2021, with some US datasets suggesting prevalence rose from approximately 1 in 10 to 1 in 5 older adults during the pandemic peak. International studies corroborate this trend, Du and Colleagues documented a 15.4% prevalence of elder abuse during the pandemic in China, with particular increases in financial abuse and neglect attributed to economic instability and social distancing measures [67]. In the European context, Filipska et al. reported an approximately six-percentage-point increase in elder abuse prevalence among hospitalized elderly patients, rising from 38.5% to 45% during the COVID-19 pandemic period [68].

These findings underscore the vulnerability of passive detection systems to disruption during public health emergencies. Systematic screening protocols, integrated into routine ED workflows and supported by electronic health record prompts, may prove more resilient to crisis-related disruptions than approaches relying on clinical suspicion [69].

Safety-planning documentation has been clarified by reporting both denominators. Overall, a safety plan was documented in 41 of 67 cases (61.2%). Among the 44 patients discharged home from the ED; however, safety planning was documented in 41 cases (93.2%).

Although most patients discharged from the ED had a documented safety plan, safety planning was not recorded in all cases. Specifically, safety plans were documented for 41 of 44 discharged patients (93.2%), whereas 3 patients (6.8%) lacked a documented safety plan. This finding should be interpreted cautiously, because the absence of a recorded safety plan may reflect incomplete documentation rather than absence of safety-oriented decision-making. In some cases, discharge may have been considered appropriate because the patient returned to an assisted-living facility, another supervised setting, or a family environment judged to be safer after ED assessment and external-agency notification. Nevertheless, incomplete documentation of safety planning remains clinically relevant, as ED discharge following suspected elder abuse should consistently include explicit assessment of the destination environment, caregiver risk, available supervision, and follow-up with social or protective services. This gap highlights a critical vulnerability in current practice. International guidelines emphasize that discharge planning for abuse victims must include comprehensive safety assessment, development of individualized safety plans, connection to community resources, and follow-up arrangements [70].

An important unresolved issue is what intervention should follow once elder abuse is identified in the ED. Screening alone is unlikely to improve outcomes unless it is linked to effective, individualized, and multidisciplinary responses. This concern is reflected in the current U.S. Preventive Services Task Force recommendation, which concludes that evidence is insufficient to assess the balance of benefits and harms of screening for caregiver abuse or neglect in older or vulnerable adults, partly because evidence on effective post-identification interventions remains limited [27]. Therefore, ED-based detection should not be interpreted as an automatic indication for institutional placement or removal from the home. Although returning home may maintain exposure to potential re-abuse, remaining in a familiar environment can also preserve autonomy, comfort, routine, and continuity of care, whereas relocation may be distressing or clinically destabilizing for some older adults. For this reason, the response to elder abuse should be individualized and should include assessment of immediate danger, decision-making capacity, cognitive status, caregiver risk, patient preferences, availability of safe caregivers, and feasibility of follow-up. In high-risk cases, hospital admission, temporary protected placement, or judicial/social-service intervention may be necessary; in other cases, a home-based safety plan with close follow-up, social-service involvement, caregiver support, and law-enforcement or judicial notification may better balance protection with autonomy. Future research should therefore evaluate not only screening tools but also the effectiveness, safety, and acceptability of different intervention pathways after ED identification of elder abuse.

Our chart review identified 19.4% of patients who returned to the ED during the study period, though documentation limitations prevented definitive determination of whether return visits were abuse-related. Prospective studies with systematic follow-up are needed to evaluate recidivism rates, intervention effectiveness, and long-term safety outcomes. The absence of such data in our retrospective study represents a significant limitation and highlights the need for prospective research with structured outcome assessment.

While the Rosa Code protocol provided a standardized response framework, our study cannot evaluate its effectiveness because all cases were identified through Rosa Code activation (i.e., no comparison group exists). The protocol’s reliance on clinical suspicion as the trigger for activation represents a fundamental limitation, as evidenced by the <1% detection rate. Future research should evaluate whether integrating validated screening tools into the Rosa Code pathway increases detection rates while maintaining the protocol’s strengths in coordinated multidisciplinary response.

Barriers to full protocol implementation should be investigated, and strategies to improve adherence developed. Potential barriers may include time constraints, staff training gaps, patient refusal, or resource limitations.

Our experience with the DA5 (Domestic Abuse, Stalking and Honour-Based Violence) risk assessment tool illustrates the challenges of applying instruments developed for intimate partner violence to elder abuse contexts. Among 28 cases after DA5 implementation, a score of 2 was the most frequent single category (39.3%) but did not represent a majority. Only one case (3.6%) received the highest score of 4. Notably, among 19 cases with documented history of previous abuse, only 4 (21.1%) had DA5 scores ≥3 (high risk), suggesting potential limitations in the tool’s sensitivity for elder abuse contexts.

The DA5 was originally designed and validated for women and especially, women victims of intimate partner violence, and its applicability to elder abuse—which involves distinct dynamics, risk factors, and perpetrator relationships—has not been established. Elder abuse risk factors include cognitive impairment, functional dependency, caregiver burden, and social isolation, which may not be adequately captured by intimate partner violence screening tools [71]. The DA-5 was administered to only a subset of the cohort ([*n*] of 67 cases), which substantially limits the generalisability of the screening findings.

The international literature from 2022 to 2026 documents significant advances in emergency department-specific screening instruments, though no single gold standard has emerged. Six tools demonstrate particular promise for ED implementation: EASI, ED Senior AID, ERASE, EM-SART, ISAR and VOICES. Each addresses different aspects of the detection challenge and offers distinct implementation advantages.

A comprehensive scoping review of nurse-administered screening tools for detecting elder abuse in emergency departments identified multiple instruments but concluded that most lack robust reliability and feasibility testing, and many are under-utilized in practice [28]. This systematic assessment underscores a critical gap: while numerous screening tools exist, rigorous multi-site validation studies establishing sensitivity, specificity, positive and negative predictive values, and inter-rater reliability remain limited. Future screening implementation in Italian EDs should prioritize elder-specific tools.

Triangulating data from multiple sources—including direct patient questioning, collateral information from caregivers, medical records, and social work assessments—is valuable as a general principle of comprehensive elder abuse assessment, not only in the context of direct questioning. Multi-source triangulation reduces reliance on victim disclosure, which is frequently inhibited by fear, cognitive impairment, or dependence on the abuser.

This study has several important limitations. First, the retrospective design limits data quality and completeness; missing or unclear documentation varied by variable and is therefore reported explicitly using available denominators and in Supplementary Table S1. Second, reliance on Rosa Code activation and clinical suspicion introduces substantial selection bias, capturing the most visible cases and precluding prevalence estimation. Third, the single-centre design may limit generalizability to smaller hospitals or rural settings. Fourth, the small sample size (*n* = 67), and especially the small number of admissions, limits statistical power for subgroup and multivariable analyses. Fifth, the absence of systematic cognitive screening likely resulted in underascertainment of cognitive impairment. Sixth, outcome data are limited, with no systematic follow-up to assess recidivism, intervention effectiveness, or long-term safety. Seventh, the study cannot evaluate the effectiveness of Rosa Code because all cases were identified through that pathway and no comparison group exists.

Despite these limitations, this study provides valuable baseline data documenting current patterns under passive case-finding and establishes the foundation for future prospective screening implementation studies.

Further research should prioritize prospective, multicentre validation of elder-specific screening tools in Italian EDs, including sensitivity, specificity, feasibility, cultural appropriateness, staff acceptability, and workflow integration. Future studies should also identify barriers and facilitators to implementation, evaluate training programmes, and assess whether screening improves patient-centred outcomes when linked to multidisciplinary safeguarding responses. Prospective follow-up is needed to measure recurrent ED presentations, re-victimization, safety-plan implementation, social-service engagement, and longer-term patient safety. Qualitative research should explore disclosure, help-seeking, autonomy, and intervention acceptability among older adults in the Italian legal and cultural context.

## 5. Conclusions

This six-year retrospective study from an Italian university hospital ED contributes to the international evidence base on elder-abuse detection in acute care settings by showing that a passive, suspicion-based pathway identified very few cases and relied entirely on Rosa Code activation. The detected cases—predominantly involving female victims, family perpetrators, home-based abuse, and physical violence with frequent head/neck injuries—align with established patterns but also highlight persistent detection gaps. All cases were identified exclusively through Rosa Code activation, confirming that passive, suspicion-based detection is the sole pathway currently in use—and that it systematically misses less visible cases.

The contemporary international literature demonstrates significant progress in developing and testing ED-specific screening tools, though rigorous multi-site validation studies remain a priority. Successful implementation requires not only validated instruments but also comprehensive training in trauma-informed approaches, multidisciplinary response teams, EHR integration, and robust linkages with adult protective services and community resources. Survivor perspectives emphasize the critical importance of rapport-building, autonomy, and individualized interventions that respect preferences and avoid re-traumatization.

Addressing elder abuse effectively demands coordinated action across multiple levels: individual clinician education and skill development, organizational policies and protocols, healthcare system integration and quality metrics, and societal commitment through legislation, funding, and public awareness. Emergency departments occupy a unique position as frontline settings where vulnerable older adults present during crises, offering critical opportunities for detection, intervention, and connection to ongoing support services.

As global populations age and the burden of elder abuse grows, healthcare systems must move beyond passive case-finding toward systematic, elder-specific ED screening linked to multidisciplinary, proportionate, and patient-centred responses. Our findings reinforce the need to integrate screening into Italian ED workflows, strengthen documentation of missing data and safety planning, train staff in elder-abuse recognition, and adapt international tools to the Italian legal and healthcare context.

## Figures and Tables

**Table 1:** Types of abuse documented in the study cohort (*n* = 67).

Type of Abuse	*n*	%
Physical abuse	41	61.2
Psychological/emotional abuse	16	23.9
Financial abuse/exploitation	6	9.0
Neglect	4	6.0
Sexual abuse	0	0.0
Abuse type not specified	15	22.4
Cases involving multiple types of abuse	10	14.9

Notes: Abuse categories were not mutually exclusive, as more than one type of abuse could be documented in the same patient. Percentages were calculated using the entire cohort as the denominator (*n* = 67) and therefore do not sum to 100%. “Abuse type not specified” indicates cases in which elder abuse was suspected or confirmed, but the available documentation did not permit classification into a specific category. The row “Cases involving multiple types of abuse” is a summary measure and should not be added to the individual abuse categories.

**Table 2:** Anatomical distribution of injuries.

Body Region	*n*	%
Head and neck	29	43.3
Upper extremities	21	31.3
Lower extremities	11	16.4
Thorax/chest	4	6.0
Other regions *	6	9.0

* Other regions include: abdomen, pelvis, and back. Note: Some victims had injuries to multiple body regions; percentages calculated from total sample.

**Table 3:** DA5 risk assessment scores (cases from April 2018 onwards, *n* = 28).

DA5 Score	*n*	%	Risk Level
0–1	10	35.7	Minimal-Low
2	11	39.3	Moderate
3	6	21.4	High
4	1	3.6	Very High
**Total**	**28**	**100.0**	—
**Mean score ± SD**	**1.9 ± 1.0**	—	—

Note: DA5 (Domestic Abuse, Stalking and Honour-Based Violence) risk assessment tool was implemented in April 2018. The DA5 findings should therefore be interpreted cautiously. The tool was available only for a limited subset of the cohort, and it was developed primarily for intimate partner violence rather than specifically for elder abuse. Accordingly, the DA5 results are presented descriptively and should not be interpreted as a validated estimate of re-victimization risk in this population. Bold formatting is used to highlight the total values.

**Table 4:** Comparison with international emergency department elder abuse studies.

Study	Country	Study Period	Sample Size (N)	Detection Method	Detection Rate	Screening Tool	Reference
Current study	Italy	2017–2022	67 cases identified from ~8371 older adults	Rosa Code (clinical suspicion)	0.8%	Clinical judgement	—
Gagnon, 2023	Canada	2021	1061	Direct questioning	5.1% (54 cases)	Direct questioning	[40]
Riedel, 2024	Switzerland	2022	1010	Systematic screening	2.9% (29 cases)	German ED Senior AID	[41]
Makaroun, 2023	USA (VA)	2020–2022	251	Systematic screening	3.6% prescreen; 2.0% comprehensive	Adapted EM-SART	[27]
Papa, 2022	USA	2020	4588	Systematic screening	0.41% (19 cases)	EMED Toolkit/EM-SART	[42]
Plummer, 2022	USA	2022	1218	Systematic screening	2.0% suspected (23 cases)	EM-SART	[43]
Richmond, 2020	USA	2020	276	Systematic screening	6.5% (18 cases)	ED Senior AID + AMT4	[44]
Platts-Mills, 2018	USA	2018	259	Tool development	7.0% (17 cases)	ED Senior AID (development)	[15]
Evans, 2017	USA	2006–2012	6,723,667 visits (weighted: 29,056,673)	Administrative coding	0.013% (3846 cases)	ICD-9-CM codes	[45]
Eulitt, 2014	USA	2014	180	Risk screening	46% at-risk (82 cases)	Modified ISAR	[46]
Fulmer, 2004	USA	2005	405	Expert adjudication	21.2% (86 neglect cases)	NAT adjudication	[47]

ED Senior AID = Emergency Department Senior Abuse Identification; EM-SART = Elder Mistreatment Screening and Response Tool; EMED = Elder Mistreatment Emergency Department; VA = Veterans Affairs; AMT4 = Abbreviated Mental Test 4; ISAR = Identification of Seniors at Risk; NAT = Neglect Assessment Team.

## Data Availability

The original contributions presented in this study are included in the article/Supplementary Material. Further inquiries can be directed to the corresponding author.

## Supplementary Materials

The following supporting information can be downloaded at https://www.mdpi.com/article/10.3390/geriatrics11040079/s1, Table S1: Documentation completeness for key study variables; Table S2: Exploratory multivariable analysis of factors associated with hospital admission.

## References

[1] World Health Organization (WHO) World Report on Ageing and Health. https://www.who.int/news-room/fact-sheets/detail/ageing-and-health.

[2] United Nations, Department of Economic and Social Affairs, Population Division (2020). World Population Ageing 2020 Highlights: Living Arrangements of Older Persons.

[3] Kitaw T.A., Baylie A., Tekle B.A., Tilahun B.D., Getie A., Abate B.B., Feleke S.F., Zemariam A.B., Azmeraw M., Yilak G. (2025). Elder abuse without borders: A systematic review and meta-analysis. BMC Public Health.

[4] Yon Y., Mikton C.R., Gassoumis Z.D., Wilber K.H. (2017). Elder abuse prevalence in community settings: A systematic review and meta-analysis. Lancet Glob. Health.

[5] Mikton C., Beaulieu M., Yon Y., Cadieux Genesse J., St-Martin K., Byrne M., Phelan A., Storey J., Rogers M., Campbell F. (2022). PROTOCOL: Global elder abuse: A mega-map of systematic reviews on prevalence, consequences, risk and protective factors and interventions. Campbell Syst. Rev..

[6] World Health Organization Elder Abuse. https://www.who.int/news-room/fact-sheets/detail/elder-abuse.

[7] Ballentine N.H. (2023). Abuse and neglect of older adults: A brief overview. GeroPsych.

[8] Lachs M.S., Williams C.S., O’Brien S., Pillemer K.A., Charlson M.E. (1998). The mortality of elder mistreatment. JAMA.

[9] Dong X., Simon M.A., Gorbien M., Percak J., Golden R. (2007). Loneliness in older chinese adults: A risk factor for elder mistreatment. J. Am. Geriatr. Soc..

[10] Acierno R., Hernandez M.A., Amstadter A.B., Resnick H.S., Steve K., Muzzy W., Kilpatrick D.G. (2010). Prevalence and correlates of emotional, physical, sexual, and financial abuse and potential neglect in the United States: The National Elder Mistreatment Study. Am. J. Public. Health.

[11] Sirey J.A., Minor M., Berman J., Geffner R., White J.W., Hamberger L.K., Rosenbaum A., Vaughan-Eden V., Vieth V.I. (2020). Intersectionality of Elder Abuse and Mental Health Issues: Needs and Interventions for Victims. Handbook of Interpersonal Violence and Abuse Across the Lifespan.

[12] Shen Y., Sun F., Feng Y., Lichtenberg P.A., Meng H. (2025). Prevalence of Elder Abuse and Neglect of Persons with Dementia in Community Settings: A Systematic Review and Meta-Analysis. Gerontology.

[13] Gurvich T., Lang A. (2023). Geriatric Pharmacotherapy Case Series: The Pharmacist’s Role in Preventing Elder Abuse. Sr. Care Pharm..

[14] Rosen T., Platts-Mills T.F., Fulmer T. (2020). Screening for elder mistreatment in emergency departments: Current progress and recommendations for next steps. J. Elder. Abus. Negl..

[15] Platts-Mills T.F., Dayaa J.A., Reeve B.B., Krajick K., Mosqueda L., Haukoos J.S., Patel M.D., Mulford C.F., McLean S.A., Sloane P.D. (2018). Development of the Emergency Department Senior Abuse Identification (ED Senior AID) tool. J. Elder. Abuse Negl..

[16] Giraldo-Rodríguez L., Agudelo-Botero M. (2022). Barriers to managing elder abuse in primary care services: Experiences of healthcare providers in Mexico City. J. Elder. Abus. Negl..

[17] Kennedy R.D. (2005). Elder abuse and neglect: The experience, knowledge, and attitudes of primary care physicians. Fam. Med..

[18] Schmeidel A.N., Daly J.M., Rosenbaum M.E., Schmuch G.A., Jogerst G.J. (2012). Health Care Professionals’ Perspectives on Barriers to Elder Abuse Detection and Reporting in Primary Care Settings. J. Elder. Abus. Negl..

[19] Hoover R.M., Polson M. (2014). Detecting Elder Abuse and Neglect: Assessment and Intervention. Am. Fam. Physician.

[20] Lachs M.S., Pillemer K.A. (2015). Elder Abuse. N. Engl. J. Med..

[21] Dong X.Q. (2015). Elder Abuse: Systematic Review and Implications for Practice. J. Am. Geriatr. Soc..

[22] Morrissey M.B., Daichman L.S., Perel-Levin S., Brownell P., Somers S.B. (2024). Elder abuse. The Cambridge Handbook of Aging and Human Rights.

[23] Ahmad S., Stern M.E., Rosen T. (2023). Clinical assessment and documentation of elder abuse. Elder Abuse: Research, Practice and Policy.

[24] Costello E.C.R. (2024). A global examination of law and the abuse of older people. International Handbook on Elder Abuse.

[25] Stoeckle R., Haggerty K.L., Fulmer T. (2022). The elder mistreatment emergency department toolkit: Addressing elder mistreatment during covid-19 and beyond. Innov. Aging.

[26] Haggerty K.L., Stoeckle R., Dash K., Epstein-Lubow G., Campetti R., Froberg R., Tambor E. (2023). The emed toolkit: Collaboration to improve elder mistreatment screening and response. Innov. Aging.

[27] Makaroun L.K., Halaszynski J.J., Rosen T., Haggerty K.L., Blatnik J.K., Froberg R., Elman A., Geary C.A., Hagy D.M., Rodriguez C. (2023). Leveraging VA geriatric emergency department accreditation to improve elder abuse detection in older Veterans using a standardized tool. Acad. Emerg. Med..

[28] El Hussein M., Sheehan D. (2025). Nurse-Administered Screening Tools for Detecting Elder Abuse in Emergency Departments: A Scoping Review. J. Adv. Nurs..

[29] Soares J., Barros H., Torres-Gonzalez F., Ioannidi-Kapolou E., Lamura G., Lindert J., de Dios Luna J., Macassa G., Melchiorre M.G., Stankunas M. (2010). Abuse and Health in Europe.

[30] Italian Penal Code, Article 365. Omissione di Referto. https://www.brocardi.it/codice-penale/libro-secondo/titolo-iii/capo-i/art365.html.

[31] Armitage R., Nellums L.B. (2020). COVID-19 and the consequences of isolating the elderly. Lancet Public Health.

[32] Krendl A.C., Perry B.L. (2021). The Impact of Sheltering in Place During the COVID-19 Pandemic on Older Adults’ Social and Mental Well-Being. J. Gerontol. B Psychol. Sci. Soc. Sci..

[33] Uzoho I.C., Baptiste-Roberts K., Animasahun A., Bronner Y. (2023). The Impact of COVID-19 Pandemic on Intimate Partner Violence (IPV) Against Women. Int. J. Soc. Determ. Health Health Serv..

[34] Fang B., Yan E. (2018). Abuse of Older Persons with Dementia: A Review of the Literature. Trauma Violence Abus..

[35] Regione Toscana Codice Rosa: Percorso per le Vittime di Violenza. https://www.regione.toscana.it/-/codice-rosa.

[36] Focardi M., D’Onofrio P., Cestaro M., Guerini M., Ermini F.R., Carnevali M., Grifoni R., Gualco B., Bianchi I., Pinchi V. (2026). Male Victims of Domestic Violence: Clinical and Behavioral Insights from an Italian Hospital-Based Study. Behav. Sci..

[37] Italian Penal Code, Article 361–362. Omissione di Rapporto. https://www.brocardi.it/codice-penale/libro-secondo/titolo-iii/capo-i/art361.html.

[38] National Research Council (2003). Elder Mistreatment: Abuse, Neglect, and Exploitation in an Aging America.

[39] Campbell J.C., Webster D.W., Glass N. (2009). The danger assessment: Validation of a lethality risk assessment instrument for intimate partner femicide. J. Interpers. Violence.

[40] Gagnon M., Dufour I., Raîche M., Côté L., Pelletier M.È., Sirois M.J. (2023). Prevalence and predictors of elder abuse among older adults attending emergency departments: A prospective cohort study. Can. J. Emerg. Med..

[41] Riedel H.B., Espejo T., Dreher-Hummel T., Bingisser R., Nickel C.H. (2024). Screening for elder mistreatment in a Swiss emergency department: A prospective cohort study. Swiss Med. Wkly..

[42] Papa C.L., Campetti R., Froberg R., Haggerty K.L., Dash K. (2022). Implementation of the elder mistreatment emergency department toolkit at heywood hospital. Innov. Aging.

[43] Plummer S., Burnett J., Froberg R., Campetti R., Dash K. (2022). Implementation of the elder mistreatment emergency department toolkit at lyndon B. Johnson hospital. Innov. Aging.

[44] Richmond N.L., Zimmerman S., Reeve B.B., Dayaa J.A., Davis M.E., Bowen S.B., Iasiello J.A., Stemerman R., Shams R.B., Haukoos J.S. (2020). Ability of Older Adults to Report Elder Abuse: An Emergency Department-Based Cross-Sectional Study. J. Am. Geriatr. Soc..

[45] Evans C.S., Hunold K.M., Rosen T., Platts-Mills T.F. (2017). Diagnosis of elder abuse in U.S. emergency departments. J. Am. Geriatr. Soc..

[46] Eulitt P.J., Tomberg M., Cunningham T.D., Counselman F.L., Palmer R.M. (2014). Screening elders in the emergency department at risk for mistreatment: A pilot study. J. Elder. Abuse Negl..

[47] Fulmer T., Guadagno L., Bitondo Dyer C., Connolly M.T. (2004). Progress in elder abuse screening and assessment instruments. J. Am. Geriatr. Soc..

[48] Corradini F., Cabiati E. (2026). Abuse against older adults and social work interventions: Where do we stand? A scoping review to explore it from an international perspective. Eur. Social. Work. Res..

[49] Mercier É., Nadeau A., Brousseau A.A., Émond M., Lowthian J., Berthelot S., Costa A.P., Mowbray F., Melady D., Yadav K. (2020). Elder Abuse in the Out-of-Hospital and Emergency Department Settings: A Scoping Review. Ann. Emerg. Med..

[50] Piquero A.R., Jennings W.G., Jemison E., Kaukinen C., Knaul F.M. (2021). Domestic violence during the COVID-19 pandemic—Evidence from a systematic review and meta-analysis. J. Crim. Justice.

[51] Gosangi B., Park H., Thomas R., Gujrathi R., Bay C.P., Raja A.S., Seltzer S.E., Balcom M.C., McDonald M.L., Orgill D.P. (2021). Exacerbation of Physical Intimate Partner Violence during COVID-19 Pandemic. Radiology.

[52] Rosen T., Hargarten S., Flomenbaum N.E., Platts-Mills T.F. (2016). Identifying Elder Abuse in the Emergency Department: Toward a Multidisciplinary Team-Based Approach. Ann. Emerg. Med..

[53] Hancock D.W., Haussner W., Chang E.I., Barghout R., Lachs J., Lees Haggerty K., Cannell B., Zhang S.X., Daniels B., Stern M. (2025). Elder Mistreatment Documentation by Prehospital Clinicians: An Analysis of the National Emergency Medical Services Information System Database. Prehosp. Emerg. Care.

[54] Yonashiro-Cho J., Gassoumis Z.D., Wilber K.H., Homeier D.C. (2021). Improving forensics: Characterizing injuries among community-dwelling physically abused older adults. J. Am. Geriatr. Soc..

[55] Kennedy C., Will J. (2021). Interventions for preventing abuse in the elderly. Int. J. Nurs. Pract..

[56] Pillemer K., Burnes D., Riffin C., Lachs M.S. (2016). Elder abuse: Global situation, risk factors, and prevention strategies. Gerontologist.

[57] Nemati-Vakilabad R., Khalili Z., Ghanbari-Afra L., Mirzaei A. (2023). The prevalence of elder abuse and risk factors: A cross-sectional study of community older adults. BMC Geriatr..

[58] Bujarad F., Edwards C., Neugroschl J.A., Hwang U., Marottoli R. (2025). Digital Elder Abuse Intervention for Early Detection of Abuse in Older Adults Living with Dementia. Alzheimer’s Dement..

[59] Friedman L.S., Avila S., Tanouye K., Joseph K. (2011). A case-control study of severe physical abuse of older adults. J. Am. Geriatr. Soc..

[60] Laumann E.O., Leitsch S.A., Waite L.J. (2008). Elder mistreatment in the United States: Prevalence estimates from a nationally representative study. J. Gerontol. B Psychol. Sci. Soc. Sci..

[61] Yan E., Chan K.L., Tiwari A. (2015). A systematic review of prevalence and risk factors for elder abuse in Asia. Trauma Violence Abus..

[62] Melchiorre M.G., Chiatti C., Lamura G., Torres-Gonzales F., Stankunas M., Lindert J., Ioannidi-Kapolou E., Barros H., Macassa G., Soares J.F. (2013). Social support, socio-economic status, health and abuse among older people in seven European countries. PLoS ONE.

[63] Rodriguez M.A., Wallace S.P., Woolf N.H., Mangione C.M. (2006). Mandatory reporting of elder abuse: Between a rock and a hard place. Ann. Fam. Med..

[64] Boserup B., McKenney M., Elkbuli A. (2020). Alarming trends in US domestic violence during the COVID-19 pandemic. Am. J. Emerg. Med..

[65] Koma W., True S., Biniek J.F., Garfield R., Claxton G., Rae M., Michaud J., Neuman T. (2020). One in Four Older Adults Report Anxiety or Depression Amid the COVID-19 Pandemic. Kaiser Family Foundation.

[66] Chang E.S., Levy B.R. (2021). High Prevalence of Elder Abuse During the COVID-19 Pandemic: Risk and Resilience Factors. Am. J. Geriatr. Psychiatry.

[67] Du P., Chen Y. (2021). Prevalence of elder abuse and victim-related risk factors during the COVID-19 pandemic in China. BMC Public Health.

[68] Filipska K., Biercewicz M., Wiśniewski A., Jabłońska R., Królikowska A., Główczewska-Siedlecka E., Kędziora-Kornatowska K., Ślusarz R. (2021). High Rate of Elder Abuse in the Time of COVID-19-A Cross Sectional Study of Geriatric and Neurology Clinic Patients. J. Clin. Med..

[69] Kayser J., Morrow-Howell N., Rosen T.E., Skees S., Doering M., Clark S., Hurka-Richardson K., Bin Shams R., Ringer T., Hwang U. (2021). Research priorities for elder abuse screening and intervention: A Geriatric Emergency Care Applied Research (GEAR) network scoping review and consensus statement. J. Elder. Abus. Negl..

[70] Heron R.L., Eisma M.C. (2021). Barriers and facilitators of disclosing domestic violence to the healthcare service: A systematic review of qualitative research. Health Soc. Care Community.

[71] Daly J.M., Merchant M.L., Jogerst G.J. (2011). Elder abuse research: A systematic review. J. Elder. Abus. Negl..

