# Supplementary material for: Detecting Elder Abuse in an Italian Emergency Department: A Six-Year Retrospective Study and Implications for Systematic Screening
Source: Geriatrics (Basel). 2026 Jul 2;11(4):79. doi: 10.3390/geriatrics11040079 (PMC13398166; doi:10.3390/geriatrics11040079)
Supplement: Supplementary file 1 [file geriatrics-11-00079-s001.zip › geriatrics-4275498-supplementary.pdf]

Supplementary Table S1. Documentation completeness for key study variables

Domain	Key variable	Available denominator, n	Missing/unclear records, n	Completeness, %
Demographic	Age	67	0	100.0
Demographic	Sex	67	0	100.0
Demographic	Nationality	67	0	100.0
Demographic	Living situation	67	0	100.0
Demographic	Marital status	67	0	100.0
Abuse-related	Type of abuse	52	15	77.6
Abuse-related	Relationship of perpetrator to victim	64	3	95.5
Abuse-related	Location of abuse	51	16	76.1
Abuse-related	Abuse chronicity (acute/isolated vs chronic/ongoing)	50	17	74.6
Abuse-related	History of previous abuse	50	17	74.6
Clinical/ED	Mode of arrival to the ED	67	0	100.0
Clinical/ED	Chief complaint	67	0	100.0
Clinical/ED	Presence or absence of visible external injury	67	0	100.0
Clinical/ED	Specific injury type documented	46	21	68.7
Clinical/ED	ED disposition	67	0	100.0
Safeguarding	Law-enforcement/police notification	67	0	100.0
Safeguarding	Referral to social services	67	0	100.0
Safeguarding	Referral to an anti-violence centre	67	0	100.0
Safeguarding	Referral to adult protective services	67	0	100.0
Safeguarding	Documented safety plan—overall cohort	41	26	61.2
Safeguarding	Documented safety plan—patients discharged home	41	3	93.2
Risk assessment	DA5 availability in the full cohort	28	39	41.8

Available denominator indicates the number of records with a usable value. Completeness was calculated as available denominator divided by the relevant cohort denominator × 100.

a. The full analytic cohort was N = 67. For the discharged-home safety-plan row, the relevant subgroup denominator was n = 44.

b. “No previous abuse documented” was treated as an available negative value; only records explicitly classified as “not documented/unclear” were counted as missing.

c. DA5 was introduced in April 2018. The 39 earlier cases were structurally not applicable rather than conventionally missing.

Supplementary Table S2. Exploratory multivariable analysis of factors associated with hospital admission.

Variable	n	aOR	95% CI	p-value
Severe injury (ref: mild)	6	138.48	5.41–3545.00	0.006**
Cognitive impairment	6	32.68	1.12–953.39	0.086†
Age ≥85 yr (ref: 65–74 yr)	7	23.05	0.80–661.34	0.134

aOR = adjusted Odds Ratio; CI = Confidence Interval
***p<0.01; †p<0.10 (trend)*

Note : Only six hospital admissions occurred. Accordingly, adjusted estimates are unstable and should be interpreted cautiously. The analysis was exploratory and was not intended to establish independent predictors of admission.
